# Evaluation of enamel surface after interproximal reduction using different methods, with and without polishing: an in vitro study

**DOI:** 10.1038/s41598-026-46967-z

**Published:** 2026-04-13

**Authors:** Lamiaa Mohammad Omar, Reham Ibrahim El Gazzar, Mona A. Montasser

**Affiliations:** 1 Department of Orthodontics, Faculty of Dentistry, Mansoura University, Mansoura, 35516 Egypt; 2 Horus University in Egypt, New Damietta, Egypt

**Keywords:** Enamel, Interproximal reduction, Surface roughness, Elements concentrations, Chemistry, Materials science

## Abstract

To evaluate the changes in enamel surface roughness and elemental concentrations following interproximal reduction (IPR), with and without polishing, in comparison with enamel without stripping. Selected premolars were randomly divided into a control group and four experimental groups; Group A (control): enamel without stripping, Group B: IPR with diamond bur, Group C: IPR with diamond disc, Group D: IPR with manual strip, Group E: IPR with mechanical oscillating strip. Each experimental group was divided into two subgroups where one subgroup was also polished with fine Sof-Lex discs. A total of 108 enamel samples (12 samples/subgroup and 12 samples for the control group) were used. Enamel was evaluated with atomic force microscope, energy-dispersive x-ray spectroscopy, and scanning electron microscope. The IPR produced higher [average roughness (nm)] in all groups compared with the enamel without stripping which measured 79.5 ± 17.4 nm. The only significant difference among the experimental groups was the higher roughness in the disc group compared to the oscillating strip group, *p* = 0.035. Polishing produced smoother enamel surfaces in all groups.The Ca/P weight (%) was 1.68 ± 0.45% when the enamel was not stripped and the ratio ranged from 2.03 ± 0.69% to 2.58 ± 0.47% in the IPR/IPR+polishing groups. Scanning electron microscope confirmed an increase in surface roughness following IPR with subsequent improvement after polishing. The IPR increased enamel surface roughness and affected the elements weight (%) and atomic (%). Polishing improved the surface quality. The IPR with oscillating strip followed by polishing with Sof-Lex strip produced the most favorable results.

## Introduction

The concept of interproximal reduction (IPR) emerged as early as 1944 as a successful approach to managing discrepancies in tooth size^1^. Approximately 50% of the enamel thickness could be stripped, in both adults and adolescents, which creates 6–8 mm in the buccal segment and 2–3 mm in the anterior area^2–4^.

The IPR procedure became an established orthodontic treatment solution that can be used as a solution for late mandibular crowding, gaining space in non-extraction cases, enhancing stability and avoiding relapse, and reshaping teeth^5,6^ and it also gained special popularity in clear aligner treatments^7,8^. However, orthodontists had concerns about possible iatrogenic effects following IPR such as caries, periodontal diseases, and tooth hypersensitivity^5^. Preserving enamel and maintaining periodontal health are in the focus of dentistry and usually achieved through oral hygiene measures and the use of protective measures^9,10^.

Several previous studies^11–13^ found an increase in surface roughness following IPR recommending polishing for smoothing the stripped enamel surfaces. In this context, it is recommended to minimize enamel surface roughness following IPR as it was found that roughness of 0.2 μm is a threshold point below it no decrease in bacterial accumulation should be expected while above it an increase in plaque accumulation should be expected increasing the risk for both caries and periodontal inflammation^14,15^.

Numerous IPR systems have been developed over the years, ranging from conventional hand-held abrasive strips, tungsten carbide or diamond burs, other powered systems such as mechanical oscillating disks or strips to bespoke developed instruments for specific use^12,16,17^.

This study aimed to evaluate changes in enamel surface roughness and elemental concentrations following IPR, with and without polishing, in comparison with enamel without stripping.

The null hypotheses were that no statistically significant differences would be observed in (1) enamel surface roughness and (2) enamel elemental concentrations following IPR or IPR followed by polishing, irrespective of the IPR method used.

## Materials and methods

### Ethical consideration

This research received approval (A0108024 OR) from the Research Ethics Committee of Faculty of Dentistry - Mansoura University. This study did not include humans or human identifiers. Informed consent was obtained from all patients prior to the collection of teeth, extracted for periodontal and/or orthodontic reasons, permitting their use for research purposes. The teeth were handled according to the biosafety guidelines.

### Sample size calculation

The sample size calculation was conducted using G* Power Software (version 3.1.9.7)^18^. Based on previous article by Danesh et al.^13^, it was hypothesized a statistically significant difference in roughness values among the 8 subgroups and the control group with large effect size (Cohen’s *f* = 0.4) i.e. a decrease of the mean roughness in all groups compared to the control group. A total of 108 enamel samples (12 samples/subgroup and 12 samples for the control group) would achieve 83% power to detect differences among the means, contrasting with the null hypothesis of equal means, using an F test (One-way ANOVA) at a significance level of 0.05. The variation in the means is represented by a large effect size (Cohen’s *f* = σm / σ = 0.4).

### Teeth selection

The research included 54 freshly extracted human mandibular premolars to obtain the 108 samples. The teeth were with a normal morphology, sound crown structure, and intact interproximal enamel. Teeth exhibiting coronal surface irregularities, crack lines, fractures, carious lesions, restorative materials, hypoplasia, abrasions, white spot lesions, or fluorosis were excluded. The teeth were thoroughly cleaned from blood, deposits, debris and calculus using an ultrasonic scaler in conjunction with a generous saline solution and disinfected using a 0.1% thymol solution for 5 min and then stored in distilled water.

### Teeth mounting

To replicate intraoral stripping conditions and for ease of handling during all procedures, the teeth were secured in typodonts (Dental Nissin, Sino Dental Group, Japan) with the crowns adjusted to remain visible 1–2 mm coronally from the cemento-enamel junction. To generate a certain amount of movement of the teeth in the typodonts^13^, the teeth were mounted with silicone material, specifically addition-type polyvinylsiloxane putty impression material (Dentsply/Detrey, Konstanz, Germany).

### The IPR groups

The selected teeth were randomly divided into a control group and four experimental groups; Group A (control): Enamel without stripping, Group B (bur): IPR with fine grit diamond bur, size 0.16 inch with safety-tipped non-cutting areas (Komet, Fort Mill, SC, USA), Group C (disc): IPR with single-sided diamond-coated disc (NTI-GmbH, Kahla, Germany) where 3 sizes were used sequentially; one opener (0.15 mm) and two for active IPR phase (0.20 mm and 0.30 mm), Group D (manual strips): IPR with double sided 6 mm width manual metallic electroplated abrasive strips (HORICO, Hopf, Ringleb & Co. GmbH & Cie., Berlin, Germany), Group E (mechanical oscillating strips): IPR with mechanical oscillating metallic strips (Jaintek Co. Ltd, Gyeonggi-do, Republic of Korea) attached to a specialized strip holder and low speed hand piece where 3 sizes were used sequentially; one opener (0.10 mm) and three for active IPR phase (0.20 mm, 0.30 mm, and 0.40 mm).

### Randomization, concealment, and blinding

Simple randomization was done using research randomizer at https://www.randomizer.org. Opaque envelopes were used for allocation concealment. Although the authors could not be blinded to the IPR or the polishing methods, atomic force microscope (AFM) and energy-dispersive X-ray spectroscopy (EDXS) measurements, and statistical analyses were blinded to the technicians and the biostatistician.

### The IPR and polishing procedures

In all the experimental groups, the IPR was performed by the same operator in accordance with the manufacturers’ instructions for each instrument. Both interproximal enamel surfaces of each tooth (mesial and distal) were stripped, with additional polishing carried out only on one surface.

Each tooth was measured mesiodistally using a digital caliper to ensure a standardized enamel reduction of 0.3 mm on each proximal surface. The amount of reduction was verified using an incremental thickness gauge. New IPR instrument was used for each enamel sample. Polishing was consistently completed within 20 s^19^ by using fine Sof-Lex discs (3 M dental product ESPE; Minnesota, USA) in a contra angle low speed handpiece. A new disc with adequate water spray was used for every enamel sample. For rotary instruments^19^, the diamond burs were operated at 400,000 revolutions per minute (rpm) under water cooling. The diamond-coated discs and oscillating strips were operated at 5,000 rpm, also under continuous water supply, to simulate clinical wet conditions. After completion of IPR and polishing, the samples were cleaned and stored in distilled water which was refreshed every seven days to prevent dehydration until the assessments were performed.

### Assessments

#### Enamel surface roughness

The AFM employs a sharp-tipped probe, typically less than 50 nm in diameter, to scan surface areas smaller than 100 μm. During scanning, the probe moves across the surface while maintaining a constant force, and the cantilever’s deflection is recorded to reconstruct a detailed image of the surface topography. This study used FlexAFM3 model (Nanosurf AG, Swizerland) that had a scan area of 10 × 10 μm³, a resolution of 256 × 256 data points, and a scan rate of 1 Hz. Scanning was performed in contact mode using a nano-conductive silicon probe and a Nanosurf C3000 software (Version 3.5:0.31). In the present study, the AFM provided both visual representations and quantitative assessments of surface roughness using surface average roughness (Sa).

#### Enamel surface elemental concentrations

The EDXS analysis was conducted using a spectrometer (Bruker, Berlin, Germany) attached to scanning electron microscopic (SEM) (Thermo Fisher Scientific Company, USA), equipped with a slew-window silicon drift detector and operated under high vacuum conditions. The parameters were set at an accelerating voltage of 15 kV, a beam current of 108 µA, an area scan mode of 250 μm × 220 μm, and an acquisition time of 200 s. In the present study, the analysis focused on determining the weight (%) and atomic (%) of the following elements: calcium (Ca), phosphorus (P), carbon (C), oxygen (O), and sodium (Na). Each element was identified based on its characteristic wavelength on the X*-*axis while, its relative abundance is plotted on the Y*-*axis.

#### Enamel surface morphology

The SEM uses a concentrated beam of electrons to scan across the analysis region, generating signals resulting from the interaction between the electrons and the samples’s surface. These signals provide detailed information about the surface topography. In the present study, three samples were thoroughly dried, mounted on aluminum stubs, and sputter-coated with a thin gold layer (approximately 10–15 nm) to enhance electrical conductivity. Examination was carried out at an accelerating voltage of 20 kV and 1500× magnification. A modified scoring scale^12,20^ was used to classify the enamel surface roughness; Score 0: The enamel surface is entirely free from scratches and grooves, Score 1: The surface shows minor scratches and grooves that are not highly pronounced, Score 2: The surface displays pronounced, deep furrows free from debris, and Score 3: The surface exhibits significant, sharply defined furrows accompanied by debris.

### Statistical analyses

Data was entered and analyzed using IBM-SPSS and GraphPad Prism software. Quantitative data was initially tested for normality using Shapiro-Wilk’s test. In the present study, results were considered statistically significant if *p* value was ≤ 0.05. Appropriate charts were used to graphically present the results whenever needed.

For the AFM results, descriptive statistics of the dependent variable, enamel surface roughness [average roughness (nm)], were calculated and the interaction between the two independent variables, the method of IPR and the additional polishing, was tested. Two-way ANOVA tested the effect of each of the two independent variables on the enamel surface roughness. A residual analysis was conducted to verify the assumptions associated with the two-way ANOVA. Outliers were evaluated through the examination of boxplots. Levene’s test was conducted to assess the homogeneity of variances. Pairwise comparisons were done with Bonferroni adjustment p value when indicated. Partial eta squared (η^2^) was used to quantify the statistically significant difference. The effect size was considered as small, medium, or large if the partial η^2^ was 0.01, 0.06, and 0.14, respectively.

For the EDXS results, the weight (%) and atomic (%) concentrations of main elements of the enamel were measured. One way ANOVA followed by Tukey’s HSD tests were used to detect differences between groups.

## Results

The mean AFM value for enamel surface roughness [average roughness (nm)] in the group without stripping was 79.5 ± 17.4 nm. The corresponding mean Sa values for the experimental groups are presented in Table 1. Results of Table 1 indicated no significant interaction between the two independent variables; the IPR method and the polishing step (*p* = 0.933). However, Table 2, demonstrated a statistically significant main effect for both variables on the dependent variable [average roughness (nm)], *p* = 0.036 for the IPR method and *p* < 0.001 for the additional polishing.

**Table 1:** Descriptive statistics and interaction of the IPR methods and the polishing effect on enamel surface roughness [average roughness (nm)]

Group	Subgroup	Mean	SD	F-statistic	Sig.	Partial η^2^
AFM results of Sa [average roughness (nm)]						
Bur	IPR	150.042	28.219	0.145	0.933	0.005
	IPR+Polishing	42.201	9.339			
Disc	IPR	162.409	18.723			
	IPR+Polishing	50.237	7.705			
Manual strip	IPR	158.023	30.029			
	IPR+Polishing	44.464	9.541			
Oscillating strip	IPR	148.240	20.655			
	IPR+Polishing	33.878	9.024			

SD = standard deviation. Sig. = significant at *P* ≤ 0.05. Partial η^2^ = Partial eta squared (a measure of effect size).

**Table 2:** Two-way ANOVA to assess the main effects for the IPR method and the polishing effect on the enamel surface roughness [average roughness (nm)]

	Mean	SE	F-statistic	Sig.	Partial η^2^
AFM results of Sa [average roughness (nm)]					
Group					
Bur	96.122	3.815	2.968	0.036	0.092
Disc	106.323	3.815			
Manual strip	101.243	3.815			
Oscillating strip	91.059	3.815			
Subgroup					
IPR	154.679	2.698	861.645	< 0.001	0.907
IPR+Polishing	42.695	2.698			

SE = standard error of the estimated mean, Sig. = significant at *P* ≤ 0.05, Partial **η**^**2**^ = Partial eta squared (a measure of effect size).

**Fig. 1:**
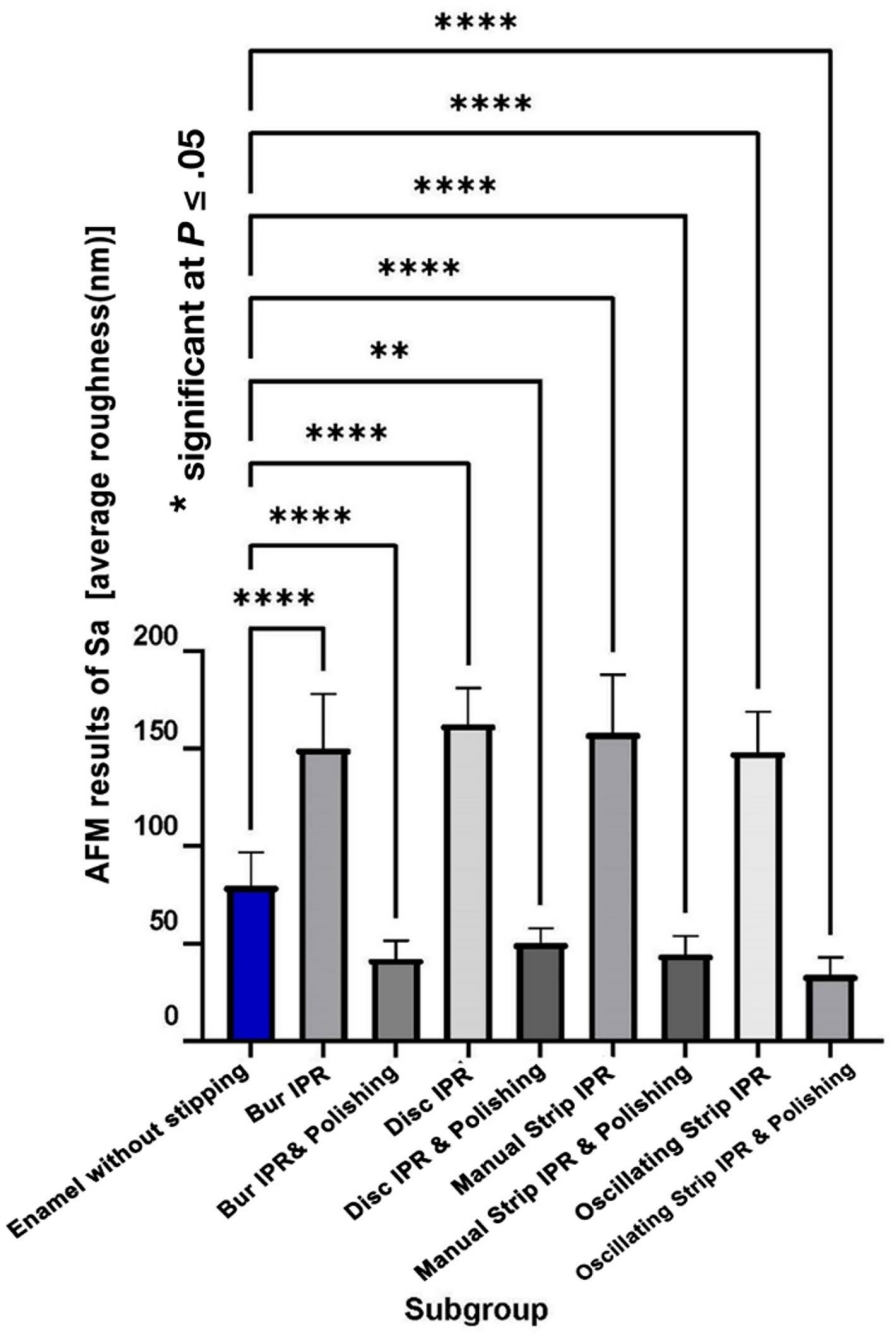
Pairwise comparisons for Sa [surface average roughness (nm)] between the enamel without stripping (control) and the enamel following IPR/IPR+polishing.

Results revealed higher [average roughness (nm)] after IPR in all groups compared with the enamel without stripping (Fig. 1). The only significant difference in enamel surface roughness among the instruments was observed between the disc and oscillating metallic strip groups, with the disc group showing higher roughness values *p* = 0.035, Table 3. Following the finishing procedure, all groups exhibited smoother enamel surfaces (Fig. 1).

**Table 3:** Pairwise comparisons between the IPR methods and the polishing on the enamel surface roughness [average roughness (nm)]

Group (I)	Group (J)	MD (I-J)	SE	Sig.^b^	95% confidence interval for difference^b^	
					Lower bound	Upper bound
Bur	Disc	− 10.201	5.395	0.372	− 24.764	4.361
	Manual strips	− 5.122	5.395	1.000	− 19.684	9.441
	Oscillating strips	5.063	5.395	1.000	− 9.500	19.625
Disc	Bur	10.201	5.395	0.372	− 4.361	24.764
	Manual strips	5.080	5.395	1.000	− 9.483	19.642
	Oscillating strips	15.264	5.395	0.035	0.701	29.826
Manual strip	Bur	5.122	5.395	1.000	− 9.441	19.684
	Disc	− 5.080	5.395	1.000	− 19.642	9.483
	Oscillating strips	10.184	5.395	0.374	− 4.379	24.747
Oscillating strip	Bur	− 5.062	5.395	1.000	− 19.625	9.500
	Disc	− 15.264	5.395	0.035	− 29.826	− 0.701
	Manual strips	− 10.184	5.395	0.374	− 24.747	4.379

Based on estimated marginal means, SE = standard error, MD = mean difference (significant at *P* ≤ 0.05), ^b^ = Adjustment for multiple comparisons: Bonferroni.

**Fig. 2:**
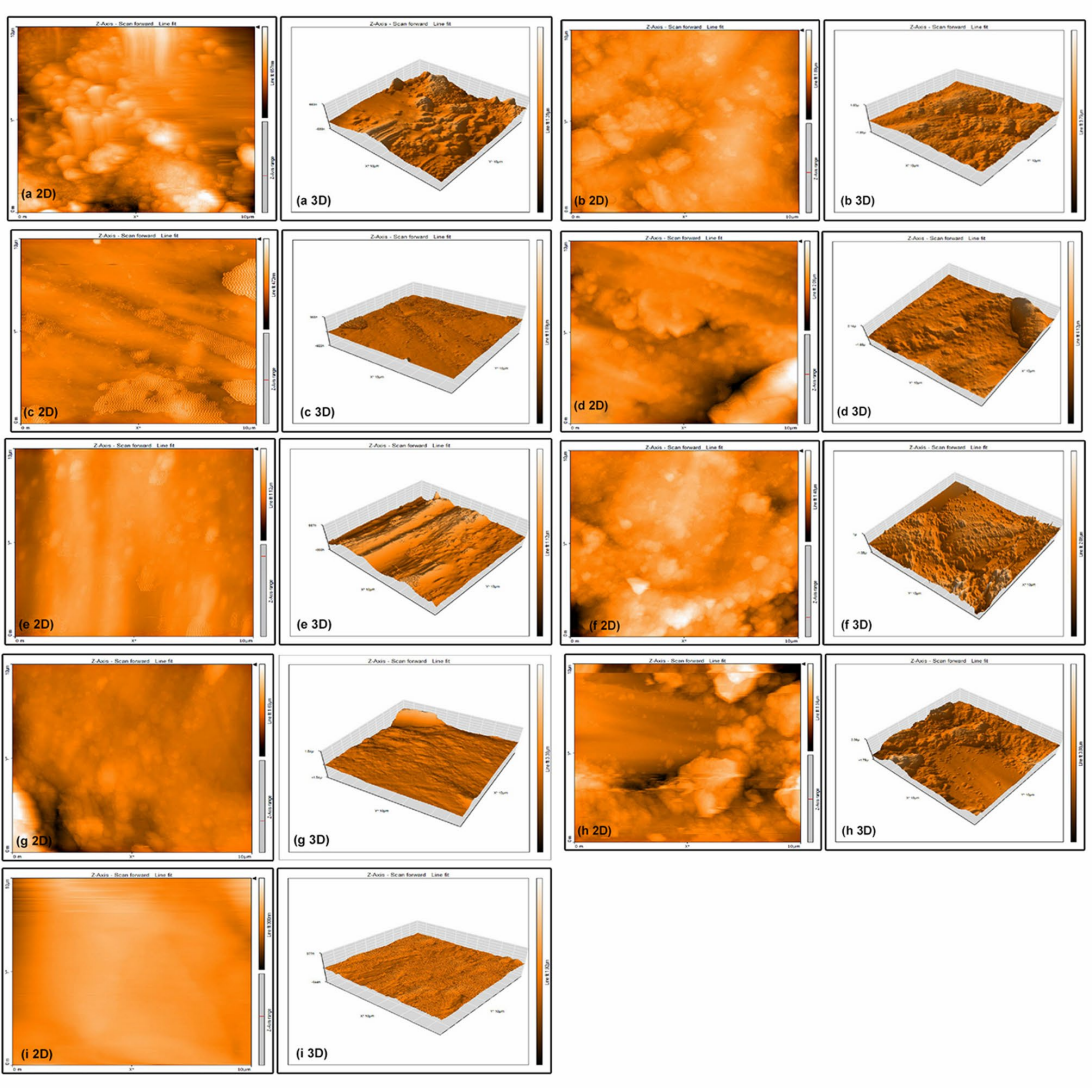
Two- and three-dimensional AFM images, (**a**) enamel without stripping, (**b**) bur IPR, (**c**) bur IPR+polishing, (**d**) disc IPR, (**e**) disc IPR+polishing, (**f**) manual strip IPR, (**g**) manual strip IPR+polishing, (**h**) oscillating strip IPR, (**i**) oscillating strip IPR+polishing.

Descriptive statistics of the elements investigated by the EDXS including the mean ± SD values are presented in Table 4. One-way ANOVA revealed statistically significant differences in all parameters studied among the groups (*P* < 0.001), Table 4. Tukey’s HSD showed that the calcium weight (%) in the enamel without stripping was (37.3 ± 5.7%) and it was not significantly different than the other groups except after IPR (36.4 ± 1.8%) with the diamond bur. However, the phosphorus weight (%) concentration was highest in the enamel without stripping (20.1 ± 1.17%) while, it was lowest after IPR with a disc followed by polishing (15 ± 3.8%) where pairwise comparisons showed significant differences between all groups. The Ca/P weight (%) was 1.68 ± 0.45% when the enamel was not stripped and the ratio ranged from 2.03 ± 0.69% to 2.58 ± 0.47% in the IPR and IPR+polishing groups (Fig. 2).

**Table 4:** One-way ANOVA comparisons of the elemental concentrations of the enamel without stripping and the enamel following IPR/IPR+Polishing.

Parameter	Control	Bur IPR	Bur IPR+Polishing	Disc IPR	Disc IPR+Polishing	Manual strip IPR	Manual strip IPR+Polishing	Oscillating strip IPR	Oscillating strip IPR+Polishing	Sig.
C weight (%)	8.4 ± 3.5	6.2 ± 1.7	6.0 ± 0.86	5.2 ± 1.8	4.6 ± 2.1	5.99 ± 2.8	5.02 ± 1.8	4.4 ± 1.4	5.1 ± 1.4	< 0.001
C atomic (%)	15.9 ± 6.3	11.6 ± 3.04	11.5 ± 1.5	10.2 ± 3.5	9.3 ± 3.9	11.8 ± 4.3	10.1 ± 3.3	8.9 ± 2.4	10.3 ± 3.9	< 0.001
O weight (%)	33.1 ± 5.3	38.06 ± 1.1	32.7 ± 4.4	40.8 ± 2.6	37.05 ± 3.2	35.7 ± 2.9	35.3 ± 4.2	37.7 ± 2.6	35.6 ± 3.3	< 0.001
O atomic (%)	41.8 ± 7.2	53.5 ± 3.1	46.2 ± 4	55.06 ± 2.7	51.4 ± 4.3	49 ± 4.2	47.3 ± 4.8	52.6 ± 3.2	50.3 ± 3.25	< 0.001
Na weight (%)	1.4 ± 0.25	1.9 ± 0.72	1.54 ± 0.85	2.45 ± 0.53	1.86 ± 0.53	2.1 ± 0.27	1.8 ± 0.47	2.24 ± 0.39	1.95 ± 0.30	< 0.001
Na atomic (%)	1.51 ± 0.23	1.9 ± 0.65	1.45 ± 0.54	2.38 ± 0.51	1.9 ± 0.6	2.1 ± 0.22	1.77 ± 0.41	2.2 ± 0.38	1.94±0.28	< 0.001
P weight (%)	20.1 ± 1.17	17.5 ± 1.32	17.1 ± 1.34	16.97 ± 1.17	15 ± 3.8	17.7 ± 1.9	17.3 ± 1.9	17.9 ± 0.84	17.5 ± 1.02	< 0.001
P atomic (%)	15.3 ± 0.69	13.1 ± 0.63	12.86 ± 0.66	12.91 ± 1.9	11.8 ± 3.9	13.6 ± 1.5	12.92 ± 1.2	13.3 ± 0.27	12.87 ± 0.64	< 0.001
Ca weight (%)	37.3 ± 5.7	36.4 ± 1.8	42.7 ± 4.4	34.5 ± 1.8	38.7 ± 3.5	38.7 ± 3.2	40.7 ± 4.6	37.96 ± 3	39.8 ± 3.85	< 0.001
Ca atomic (%)	25.5 ± 1.95	19.8 ± 2.5	27.8 ± 3.7	19.9 ± 1.7	24.2 ± 2.9	24.4 ± 3.3	26.4 ± 2.2	23.3 ± 3.2	25.4 ± 4.7	< 0.001
Ca|P weight (%)	1.68 ± 0.45	2.08 ± 0.61	2.50 ± 0.59	2.03 ± 0.69	2.58 ± 0.47	2.18 ± 0.42	2.35 ± 0.51	2.12 ± 0.58	2.27 ± 0.62	< 0.001
Ca|P atomic (%)	1.67 ± 0.45	1.5 ± 0.39	2.16 ± 0.53	1.54 ± 0.32	2.05 ± 0.54	1.79 ± 0.29	2.04 ± 0.45	1.75 ± 0.36	1.97 ± 0.39	< 0.001

Data: Mean ± Standard Deviation, Sig. = significant at *P* ≤ 0.05.

**Fig. 3:**
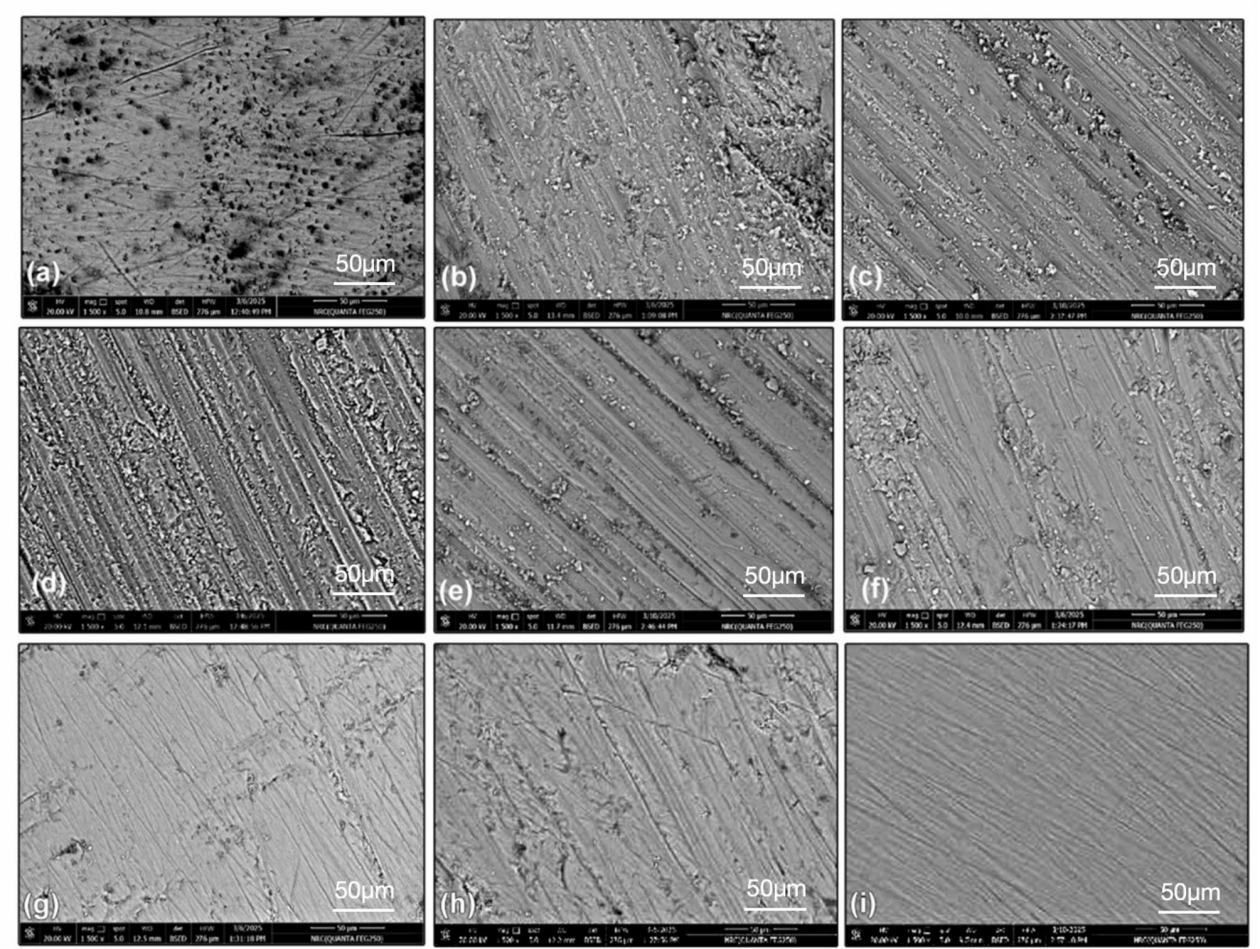
Two-dimensional 1500× SEM images, (**a**) enamel without stripping, (**b**) bur IPR, (**c**) bur IPR+polishing, (**d**) disc IPR, (**e**) disc IPR+polishing, (**f**) manual strip IPR, (**g**) manual strip IPR+polishing, (**h**) oscillating strip IPR, (**i**) oscillating strip IPR+polishing.

The SEM images representing the enamel surface without IPR (Fig. 3a) and after IPR, with or without polishing (Fig. 3b and i), revealed an increase in surface roughness following IPR, which subsequently improved after polishing. The enamel surfaces after IPR using bur, disc, and manual strips (Fig. 3b and d, and 3f, respectively) were awarded a score of 3, as each showed sharply defined furrows and scratches accompanied by visible debris. After polishing these surfaces, enamel smoothness improved, with furrows, scratches, and debris reduced but not completely eliminated (Fig. 3c, e), so the surfaces remained scored as 3. In contrast, the enamel surface after IPR with manual strips, shown in Fig. 3f, exhibited noticeable improvement after polishing (Fig. 3g) with debris disappearance and was therefore awarded a score of 2. The enamel surface after IPR using mechanical oscillating strips (Fig. 3h) displayed pronounced deep furrows free of debris and was awarded a score of 2. Polishing this surface produced a much smoother enamel, with shallow scratches and grooves that were not highly pronounced, resulting in a score of 1.

## Discussion

The findings of the present study led to the rejection of both null hypotheses. Contrary to the set hypothesis that no significant differences would be observed, the results demonstrated that enamel surface roughness was significantly affected following IPR procedures, with and without polishing. Similarly, the second null hypothesis was rejected, as statistically significant differences in enamel elemental concentrations were detected after IPR, with and without polishing.

The present study utilized premolars, as premolars extracted for orthodontic or periodontal reasons are the most commonly examined in in vitro research, thereby facilitating comparison with existing literature. The selected teeth were carefully assessed to ensure soundness and the absence of anomalies related to enamel thickness, morphology, or structure^21,22^. The IPR was performed using a diamond bur, diamond disc, manual strip, and mechanical oscillating strip. Half of the specimens subjected to IPR were subsequently polished using fine Sof-Lex discs. Enamel surface characteristics were evaluated using AFM, EDXS, and SEM to provide a comprehensive assessment of enamel surface changes. The application of these evaluation methods is well established in the literature^13,19,23,29^.

In agreement with previous studies^12,13,19^, the present study demonstrated an increase in enamel surface roughness following IPR, irrespective of the instrument used, when compared with the enamel without stripping. Despite this, the resulting surface roughness values remained below the commonly cited clinical threshold of 200 nm, above which increased plaque accumulation is expected^14,15^. Danesh et al.^13^ evaluated enamel surface roughness following IPR using various manual and mechanical instruments. In their study, one proximal surface of each tooth was subjected to IPR, while the other surface was polished after IPR. Profilometric analysis revealed varying degrees of increased surface roughness in all experimental groups depending on the instrument used. Using AFM to examine enamel surface following IPR performed with diamond burs, strips, and discs showed an increase in surface roughness with them all^19^. Among the tested instruments, diamond burs produced the roughest surfaces, followed by diamond strips and then diamond discs. Furthermore, qualitative SEM evaluation of enamel surfaces after IPR performed with three mechanical (oscillating strips) and three manual (hand-held strips) systems demonstrated less enamel debris and fewer residual abrasive particles when mechanical strips were used, indicating a smoother and more regular enamel surface compared with manual strips^12^.

Several factors may contribute to the surface roughness produced after IPR, including the type and grit size of the abrasive coating, the rotational speed of the air-rotor, and operator-related factors such as the degree of control over the IPR instrument^13,24^. In the present study, enamel surfaces stripped with burs, discs, manual strips, and oscillating strips exhibited similarly increased roughness values that were significantly higher than those of enamel without stripping. However, among the different IPR methods, a statistically significant difference was observed only between the disc and oscillating strip groups. This finding may be attributed to the reciprocating motion of mechanical oscillating instruments, which are typically operated at low speeds, allowing for more controlled enamel removal and potentially resulting in a smoother and more uniform surface.

To enhance enamel surface smoothness and eliminate residual enamel debris and abrasive particles following IPR, polishing is considered an indispensable step. Although polishing may not completely remove the scratches, furrows, and residual debris produced during the IPR procedure, several studies have reported a significant reduction in surface roughness after polishing^11,13,25^. Consequently, there is a general consensus regarding the necessity of thorough polishing after IPR. When evaluating IPR with tungsten carbide burs and diamond burs of different grit sizes, followed by polishing with Sof-Lex discs, it was concluded that the use of a tungsten carbide bur, particularly when combined with Sof-Lex disc polishing, produced a well-polished enamel surface that could be even smoother than enamel without stripping^25^. Consistent with previous reports^24,25^, increased enamel roughness following IPR makes it more difficult to achieve a smooth, furrow-free surface after polishing. In the present study, the oscillating metallic strip group demonstrated the lowest surface roughness after IPR and achieved the smoothest enamel surface following polishing, whereas groups exhibiting higher roughness following the IPR, showed less improvement in surface smoothness. Although the polishing time in the present study was standardized at 20 s^19^, this fixed duration may not accurately reflect the variable clinical requirements needed to achieve an optimal enamel surface following IPR. Individual operator-related factors may influence the effective use of the allocated polishing time. Furthermore, the optimal duration of polishing may vary depending on the degree of surface roughness produced by the IPR method employed, as well as the polishing system used.

The results of the EDXS analysis in the present study suggested that IPR affected the concentrations of the investigated elements. The analyzed elements; Ca, P, Na, O, and C are consistent with those evaluated in previous studies investigating enamel elemental changes following IPR and other mechanical surface treatments^26–28^. The principal constituents of hydroxyapatite, Ca and P, are commonly assessed as indicators of enamel demineralization and/or remineralization. This is particularly relevant given concerns that the IPR procedure may remove the superficial mineral-rich enamel layer, thereby exposing deeper layers that are more susceptible to demineralization^29^. Alterations in elemental concentrations may consequently increase susceptibility to demineralization, which clinically manifests as white spot lesions representing initial caries. In this context, a previous study^30^ evaluated the susceptibility of enamel stripped by 0.5 mm using an air-rotor instrument compared with enamel without stripping by immersing premolars in a demineralizing solution for varying periods of up to 14 days. At all evaluated time intervals, the mineral density of stripped enamel was significantly lower than that of enamel without stripping, even following fluoride application.

A Previous study^29^ evaluated the elemental concentrations of proximal enamel surfaces subjected to 0.2 mm, 0.3 mm, and 0.5 mm of IPR and extracted after one month, and reported no significant differences between the elemental concentration of enamel after IPR and enamel without stripping. However, as this was an in-vivo study, the potential occurrence of remineralization of the stripped enamel during the 1 month period after IPR cannot be excluded. In contrast, in the present study, the Ca/P weight (%) increased in all groups following IPR as well as after IPR followed by polishing. These findings contradict the results of a previous in-vivo study^31^ which reported a lower Ca/P mass (%) in enamel surfaces after IPR compared with the enamel without stripping. This discrepancy may be attributed to differences in IPR methods, polishing procedures, the amount of enamel reduction, the analytical methods used, and whether the study was conducted in vitro or in vivo. Changes in C content are often attributed to alterations in the organic component of enamel or to surface contamination following mechanical procedures. In the present study, the observed changes in the C content may be explained by reduced surface contamination resulting from the removal of the most superficial enamel layer during IPR. Additionally, an accompanying increase in Na and O mass (%) was observed in all experimental groups.

While the observed changes in the Ca/P ratio may reflect alterations induced by IPR, several methodological factors can influence the absolute values obtained and should be carefully considered. EDX is a standardless, semi-quantitative technique that provides only approximate elemental concentrations, with a higher margin of error compared to fully quantitative methods. Moreover, EDX analyzes a subsurface interaction volume rather than a single point, which may mask surface-specific changes. Surface roughness following IPR, with or without polishing, can significantly affect electron scattering and X-ray generation and propagation, thereby introducing systematic errors. In addition, specimen-related factors such as contamination or dehydration during preparation, may further influence the accuracy of the measurements^32,33^.

Qualitative assessment of the enamel surface is also essential. The SEM scans provide highly detailed images that offer readers, researchers, and clinicians clear visual representations of surface morphology, which are often easier to interpret than those obtained using other analytical methods. In the present study, SEM findings were consistent with the quantitative roughness values measured by AFM. The images demonstrated increased enamel surface roughness following IPR, which was noticeably reduced after polishing. These observations are in agreement with the SEM findings of a previous study^11^ which reported roughened and grooved enamel surfaces after the use of various stripping protocols. Polishing with Sof-Lex discs resulted in smoother enamel surfaces, although furrows, debris, and traces of the polishing procedure were still evident.

In vitro studies are inherently limited by their inability to fully replicate the complex oral environment. As the present study focused on the immediate evaluation of enamel surface following IPR and IPR combined with polishing, certain limitations should be acknowledged, particularly the absence of saliva and a true periodontal response. Saliva is a highly dilute and complex biological fluid composed of more than 99% water, in addition to components such as mucins, glycoproteins, enzymes, and other proteins that form a thin protective film on oral surfaces^34^. These constituents confer lubricating properties to saliva, significantly reducing friction between hard surfaces^35^. In the present study, such surfaces include the enamel and the IPR instrument. In vivo, the viscoelastic properties of the PDL play a critical role in absorbing and dissipating mechanical stresses applied to teeth. In contrast, in vitro models lack this physiological mechanism, which may result in altered mechanical behavior of the samples compared with clinical conditions^36^. This difference may potentially influence both the extent of enamel removal and the degree of surface damage observed. To partially mitigate these limitations, a moist environment was maintained throughout the IPR, and the teeth were mounted in polyvinyl siloxane putty material to simulate periodontal support. Lastly, While the scoring system enhanced objectivity, the small sample size prevented statistical comparison; larger samples are recommended in future studies.

## Conclusion

The IPR procedure increased enamel surface roughness and affected the elements weight (%) and atomic (%) regardless of the instrument used. However, subsequent polishing improved the surface quality. The combination of IPR using mechanical oscillating strips followed by polishing with Sof-Lex strips produced the most favorable results. Limitations of the present study should be considered.

## Data Availability

The data of this study are available from the corresponding author, upon reasonable request.
